# Progenitors of the protochordate ocellus as an evolutionary origin of the neural crest

**DOI:** 10.1186/2041-9139-4-12

**Published:** 2013-04-10

**Authors:** Evgeniy Ivashkin, Igor Adameyko

**Affiliations:** 1 Koltzov Institute of Developmental Biology, Vavilova 26, Moscow 119334, Russia; 2 Department of Medical Biochemistry and Biophysics, Karolinska Institutet, Scheeles vag 1 A1, Stockholm 17177, Sweden

**Keywords:** Neural crest, Evolution, Ocelli, Photoreception, Development

## Abstract

The neural crest represents a highly multipotent population of embryonic stem cells found only in vertebrate embryos. Acquisition of the neural crest during the evolution of vertebrates was a great advantage, providing *Chordata* animals with the first cellular cartilage, bone, dentition, advanced nervous system and other innovations. Today not much is known about the evolutionary origin of neural crest cells. Here we propose a novel scenario in which the neural crest originates from neuroectodermal progenitors of the pigmented ocelli in *Amphioxus*-like animals. We suggest that because of changes in photoreception needs, these multipotent progenitors of photoreceptors gained the ability to migrate outside of the central nervous system and subsequently started to give rise to neural, glial and pigmented progeny at the periphery.

## Background

### Introduction to neural crest biology

**Figure 1:**
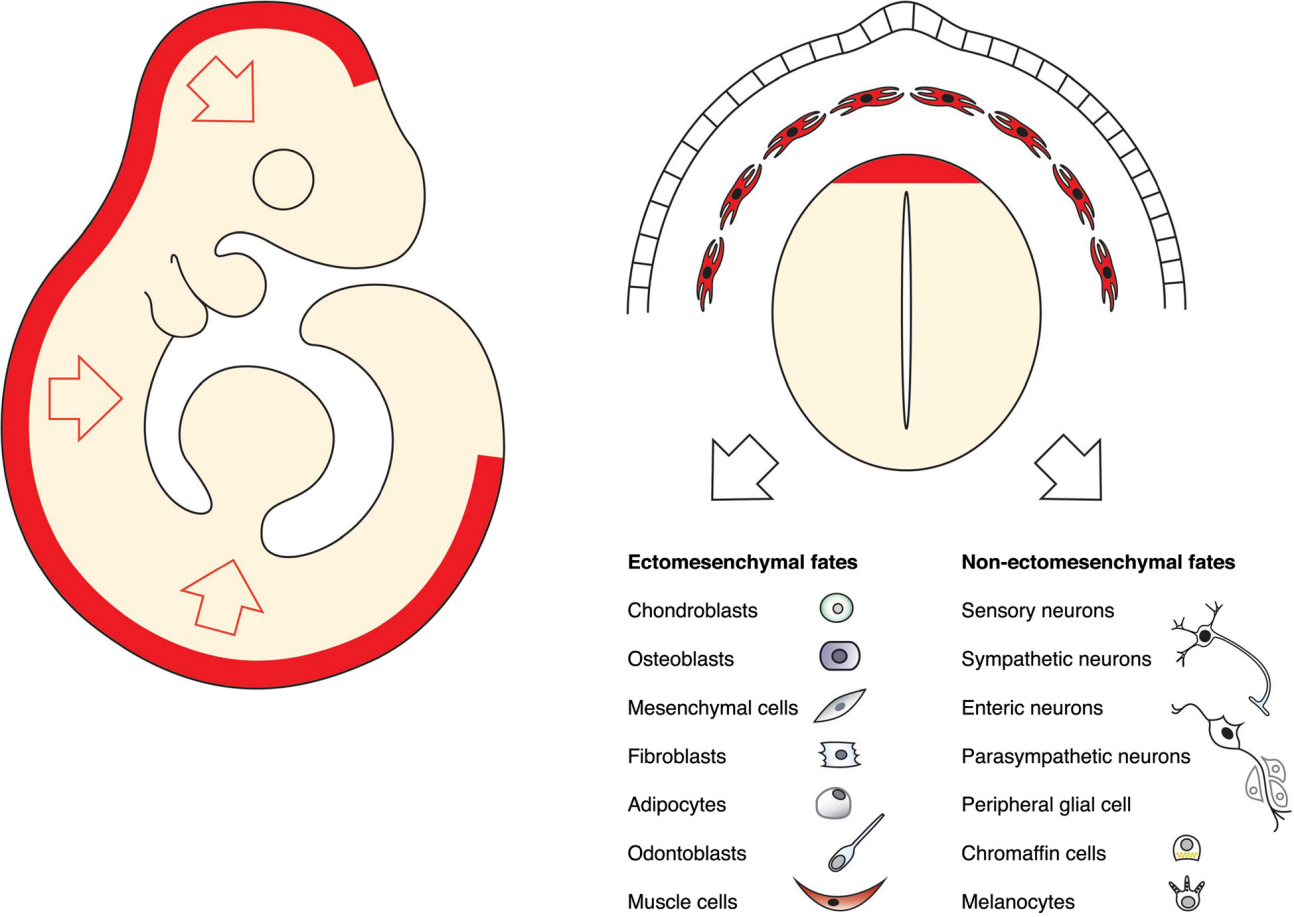
Neural crest cells are embryonic multipotent stem cells giving rise to a broad range of ectomesenchymal and non-ectomesenchymal fates.

Neural crest cells are transient embryonic multipotent migratory cells of ectodermal origin, which are unique to vertebrates. These cells are evolutionarily recent and truly essential for constructing a vertebrate body since they provide a cranial skeleton, advanced sensory systems, improved endocrine regulation, fast propagation of the action potential in the PNS, dentition, a heart outflow tract, a blood–brain barrier and pigmentation. During development neural crest cells mainly give rise to cells of two kinds: ectomesenchymal (including cartilage, bone, odontoblasts, smooth muscle, mesenchymal cells, adipocytes) and non-ectomesenchymal (neurons, glia, melanocytes, chromaffin cells) (Figure 1) [1]. A number of completely novel tissues emerged in the vertebrate lineage during neural crest evolution, for example, cellular cartilage, dentin and bone. As a result, the neural crest and cranial placodes equipped a simple *Chordata* animal with a cranium bearing powerful sensory organs. Thus, neural crest cells shaped the vertebrate body plan and provided our distant filter-feeding ancestors with advantages in predation, locomotion and an active lifestyle [2].

Development of the neural crest begins with its specification during gastrulation and continues in the dorsal neural tube, where neuroepithelial progenitors undergo an epithelial-to-mesenchymal transition and delaminate into the subepithelial space [3]. A number of inductive and specification events govern the appearance of the neural crest at the proper time and in this specific location. After delamination, neural crest cells migrate to their intermediate and final destinations using complex navigational cues and taking different routes in the body [4]. The majority of the neural crest cells stay multipotent at the very beginning of their migration, becoming increasingly restricted in their differentiation potential on their way [5,6]. However, some recent studies suggest that a part of the neural crest population might already be specified inside of the neural tube or that its specification is regulated by the timing of delamination [7,8]. Finally, if we were to describe neural crest cells in just a few words, we would say: modern, migratory and multipotent.

The Holy Grail of contemporary biology is the knowledge of principles underlying the generation of novel cell types by co-option of existing and invention of novel developmental mechanisms and cellular functions. The neural crest represents a perfect model for addressing the mechanisms behind evolutionary innovations providing opportunities to observe how multipotency evolved in a specific cellular lineage and how new cell types were eventually developed throughout different groups of animals. The emergence and evolution of the neural crest hold the key to our understanding of the evolutionary novelty on the molecular, cellular and the whole organismal levels.

### Current views on the neural crest evolution

Migratory and multipotent neural crest stem cells delaminating from the dorsal neural tube appear already in a common ancestor of gnathostomes and cyclostomes since perfectly fine neural crest cells are found in both lamprey and hagfish. Importantly, there is a difference in a range of progeny generated by the neural crest at different levels of organization. Lamprey neural crest, for instance, does not give rise to a sympathetic nervous system, bone, dentin, myelinating peripheral glial cells but forms different types of cellular cartilage in the body (soft and hard) [9-12]. Based on this, current opinion holds that elaboration of the fully multipotent neural crest population occurred incrementally through different vertebrate taxa [13,14]. However, this knowledge does not help to solve the neural crest origin dilemma. It is highly unclear if the first proto-neural crest cells were multipotential, giving rise to cells of a few different fates, or unipotential, only giving rise to a certain cell type.

Recent research revealed that at the level of cyclostomes neural crest formation is already governed by a group of evolutionarily conserved molecules organized into the so-called neural crest gene regulatory network (NC-GRN) [15]. These molecules orchestrate the formation of the neural crest in all vertebrates and include neural plate inducers and border specifiers (FGF, BMP, Wnt, Dlx, Msx1/2, Pax3/7, Zic), neural crest specifiers (Slug/Snail, FoxD3, AP-2, Twist, Id, c-Myc, members of SoxE family), neural crest delamination and migration controllers (RhoB, Cadherins), and, finally, neural crest effectors (MITF, Kit, Col2a, cRet, Erbb3) [15-17]. Currently, the concept of the NC-GRN represents a major and key advancement in our understanding of neural crest evolution and co-option of new functions [18]. Since the neural crest together with the corresponding NC-GRN appears to be fairly well conserved in all vertebrates, researchers conducted a search for expression of components of this network in lower branches of the *Chordata* phylogenetic tree, represented by the *Tunicata* and *Cephalochordata* groups.

Indeed, ascidian tunicates have a fraction of the neural crest gene regulatory network expressed in the trunk lateral cells. Those neural crest specifier genes include FoxDb, c-Myc, Twist-like 1 and 2 together with very few downstream neural crest effectors (cadherin-2, RhoABC) [19]. In line with this, a recent discovery showed that tunicates possess migratory neural crest-like cells giving rise to pigmentation. Experiments with DiI tracing in developing ascidian *Ecteinascidia turbinata* larva enabled the detection of cells migrating from the neural tube region into the body wall and the siphon where they were differentiating into pigmented cells [20]. As a result, the authors of this study hypothesized that the evolution of the neural crest started with the exit of pigmented cells from the CNS to give rise to pigmentation of the body. However, there is a criticism focused on some non-specific DiI labeling of cells adjacent to the neural tube and also on other aspects of this experiment [14,21]. William Jeffery conducted follow-up experiments with HNK-1 and tyrosinase immunohistochemistry labeling focused on 12 very diverse ascidian species to address the nature of those migratory pigmented cells. The set of analyzed ascidian species included both solitary and colonial forms, different adult organizations and developmental modes, and variation in larval sizes and complexities. It turned out that, indeed, the population of migratory pigmented cells positive for both HNK-1 and tyrosinase exists in all checked ascidian species including *Ciona intestinalis* and *Ecteinascidia turbinata*. Moreover, albino morphs of *Botryllus schlosseri* demonstrated the absence of HNK-1^+^/tyrosinase^+^ cells, which additionally confirms that HNK-1 and tyrosinase markers label pigmented cells [22]. In a follow-up publication, Jeffery and coauthors carried out accurate lineage tracing based on a cleavage arrest technique in ascidian *Ciona intestinalis*[23]. As a result, the authors revealed that migratory HNK-1^+^ neural crest-like cells originate not from CNS, but from A7.6 cells (precursors of trunk lateral cells), which belong to the mesodermal lineage and do not express Msx, Dlx, Zic and Pax genes [22,23].

Therefore, these neural crest-like cells most likely represent the result of parallel evolution rather than the true neural crest homologous tissue. This is not a surprising interpretation, since other deuterostomes, for example, sea urchins, also acquire pigment cells of mesodermal origin [24,25]. Interestingly, mesenchymal trunk lateral cells derived from A7.6 progenitors express Twist [23], which, as we suggest, could be co-opted into the neuroepithelial lineage to finally assemble migratory neural crest fate in the evolution of early chordates (see our discussion of the rudimentary neural crest in ascidians below).

Another interesting aspect of ascidian life is that the adult ascidian ganglion continues to grow and, as previously suggested, the source of the ganglion cells might be the dorsal strand – a structure derived from embryonic neural tube [26]. The ganglion can also regenerate after damage, and GnRH-immunoreactive neurons residing in the dorsal plexus appear to be the source of regenerated cells in the ganglion [26-28]. Marianne Bronner-Fraser and Clare Baker suggested that these GnRH-immunoreactive neurons may be derived from the dorsal strand. Bronner-Fraser and Baker point out that if this were the case, the precursors of these GnRH-immunoreactive neurons would be considered similar to the neural crest [26] and, we believe, deserve additional attention in regards to their status as possible neural crest homologous tissue in tunicates.

This was not the last direction in the search for the rudimentary neural crest or cells similar to the neural crest in ascidians. Some time ago, Clare Baker and Marianne Bronner-Fraser proposed that melanin-containing cells in ascidian CNS might be evolutionary precursors of neural crest-derived melanocytes [26]. Recently, Philip Barron Abitua and Michael Levine with coworkers demonstrated that tunicate *Ciona intestinalis* possesses a cephalic melanocyte lineage (a9.49) similar to the neural crest that can be reprogrammed into migratory multipotent population by introduction of Twist [29]. This lineage expresses neural plate border genes and neural crest specification genes Id, Snail, Ets and FoxD [23,29-34], and it gives rise to melanocyte of light-detecting ocellus and otolith [35]. Normally Twist is expressed only in multipotent migratory mesoderm-derived mesenchymal cells in *Ciona intestinalis*, giving rise to a number of mesodermal derivatives including body-wall muscles, tunic cells and blood cells. However, after introduction of Twist into cephalic melanocyte lineage, cells acquired neural crest-like migratory properties and produced various tissues of mesodermal origin. The authors also showed that signaling pathways governing specification of the ascidian ocellus are conserved with specification events in vertebrate neural crest lineage including Wnt signaling and FoxD-mediated repression of MITF.

There is still a possibility that the interaction between MITF and FoxD represents a very ancient module evolved well before the split of chordates and other animals (probably in photosensory structures), in this case without original connection to the neural crest. However, the conservative coinciding presence of MITF/FoxD repression, the Wnt signaling module and other neural crest-specific regulatory proteins in the ascidian-pigmented ocellus lineage and neural crest makes a plausible case for an evolutionary connection. Thus, the authors present compelling evidence that the bilateral a9.49 pigment cell lineage of tunicate embryo represents a rudimentary neural crest [29]. This study supports the hypothesis that mesenchymal properties and fates were a late acquisition during neural crest evolution [2,36]. However, it is not clear how multiple mesenchymal fates are enabled upon introduction of Twist and migration of targeted cells outside of the CNS. Specifically, we are interested in finding out what dictates these new fates – new intrinsic codes and unlocked potential of the lineage or/and extrinsic signals coming from the environment.

**Figure 2:**
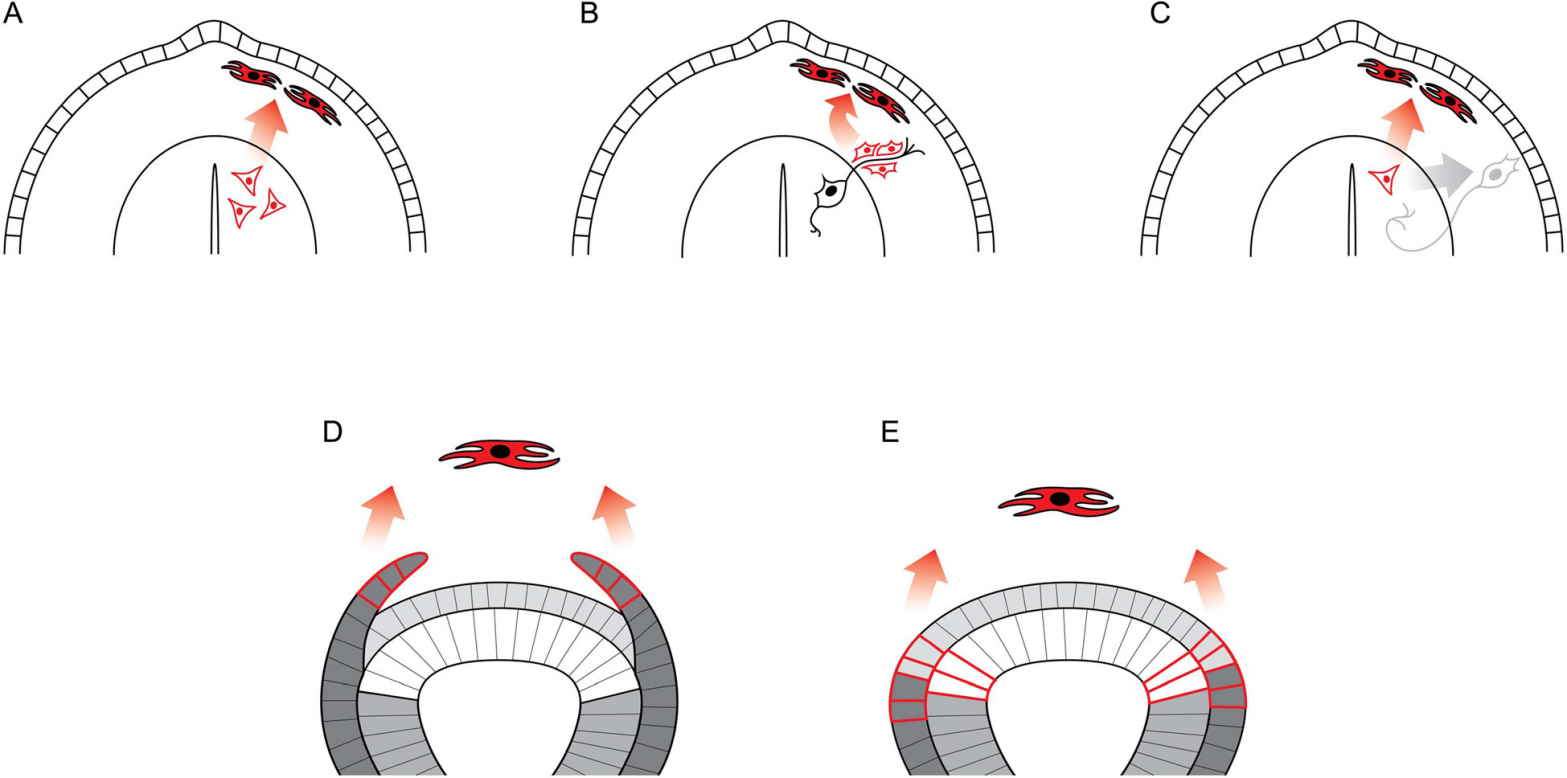
**Some suggestions made by different authors regarding the evolutionary origin of the neural crest.** Proto-neural crest cells are filled with red color, and their hypothesized origins are represented by the cells with red contours. (**A**) Evolution of the neural crest started with the exit of pigmented cells from the CNS to give rise to pigmentation of the body in lower chordates [20]. (**B**) The neural crest originated from the rare peripheral glial cells from the *Amphioxus* dorsal roots. (**C**) Precursors of Rohon-Beard sensory neurons migrated out of the neural tube and gave rise to the peripheral progeny consisting of sensory neurons and glia, and thus proto-neural crest [13,50]. (**D**) Neural crest cells originated from the overlaying non-neural ectodermal sheets appearing during *Amphioxus* development [42]. (**E**) Neural crest cells originated from several independent cell types with diverse differentiation potentials that have collectively assembled into a presumptive proto-neural crest at the neural plate border [19].

*Amphioxus* (lancelet), the most basal contemporary chordate animal, is of special interest in regard to neural crest evolution. *Amphioxus* does not have neural crest cells or any other cells delaminating from the dorsal neural tube or adjacent non-neural ectoderm. Numerous studies address the expression of neural crest gene regulatory network components in developing lancelet larva. The few genes known to be expressed in the premigratory and migratory vertebrate neural crest appear in the non-neural ectoderm of *Amphioxus*. These include *BMP2*/*4*[37], the *snail*/*slug* genes, *Pax3*/*7* genes and *Msx*[38-40]. Moreover, neurulation in *Amphioxus* occurs in a way different from neurulation in higher vertebrates and cyclostomes. The non-neural ectodermal sheets migrate above the invaginating neural plate toward the dorsal midline where they fuse. At the same time, the neural ectoderm rolls up into a tubular structure under the spanning non-neural ectoderm. However, it appears that these non-neural epithelial cells never migrate as individuals [41]. Linda and Nicholas Holland suggested that migratory non-neural ectodermal sheets might represent a homologous tissue to a vertebrate neural crest [42]. This hypothesis was additionally supported by data from previous research on developing hagfish larva, which revealed the presence of epithelial pockets adjacent to the neural ectoderm. These pockets or invaginations were interpreted as another step in neural crest evolution and as a transformation of the epithelial migratory sheets from lancelet embryo. Yet, recent success in a hagfish embryology demonstrated that the epithelial pockets turned out to be an artifact of *in toto* fixation of hagfish embryos. In fact, a hagfish, as well as a lamprey and other vertebrates, has a normally delaminating neural crest [43]. These results show that delaminating migratory neural crest cells were already present in an ancestor of the hagfish and other vertebrates in the Cambrian period approximately 500 million years ago [44]. However, unlike in cyclostomes, *Amphioxus* homologs of key neural crest specifiers other than Snail are not expressed in the neural plate [38]. Instead, c-Myc, Id, Twist, FoxD3 and SoxE are found in the mesoderm and endoderm, while AP-2 appears only in the epidermis. Additionally, expression of Id and FoxD proteins was found only in the anterior ectoderm [18,45-49]. Interestingly, some neural crest effector programs appear to be conserved in *Amphioxus* and are responsible, for example, for the production of a melanin-based pigment (*MITF*, *Trp*) [45]. Based on this, Natalya Nikitina and coworkers suggested that several independent cell types with diverse differentiation potentials might have collectively assembled into a presumptive tissue at the neural plate border, thus giving rise to the future multipotent neural crest cells (Nikitina et al., 2009). This concept is diametrically opposed to previous ideas, which were mostly proposing unipotent migratory proto-neural crest cells gaining multipotency later. One such hypothesis implies that primitive neural crest cells were eventually elaborated from progenitors of sensory neurons located inside the *Amphioxus* neural tube [13,50]. Another hypothesis suggests that the neural crest originated from the rare peripheral glial cells (or their progenitors) from the *Amphioxus* dorsal roots. These glial elements might be considered similar in some ways to vertebrate neural crest-derived Schwann cell precursors (Figure 2) [21].

Importantly, chordate outgroups including echinoderms and hemichordates have also attracted the attention of researchers in regards to neural crest origin and evolution, as reviewed by Clare Baker and Marianne Bronner-Fraser [26]. However, experimental evidence is very limited in these organisms, thus not allowing drawing any significant conclusions.

In some cases, researchers discuss the evolutionary and embryonic origins of the neural crest in conjunction with the development of cranial placodes since these tissues share many common features, such as proteoglycan secretion, multipotentiality and ectodermal origin [51,52]. In the latest review on the topic, Gerhard Schlosser concludes that there are crucial differences between these two embryonic tissues. Dissimilarities in genetic networks involved in specification, ectoderm competence states and intrinsic migratory properties support independent evolutionary and embryonic origins for cranial placodes and neural crest cells [53,54].

Finally, most current views on the neural crest evolution lean towards the very general idea that neural crest cells originated from the neural ectoderm of some primitive *Chordata* animals. Multipotency is usually seen in this case as the result of incremental evolution of the neural crest elaborating [21,26] and/or co-opting [14] additional fates with time.

Importantly, incremental evolution of neural crest fates does not mean that corresponding molecular mechanisms were always co-opted from other tissues. Co-option of the gene regulatory network stands for the shift of expression of several key players (forming a stereotypical gene regulatory network) to a new location. Indeed, some authors suggested that, for instance, mesenchymal fates were co-opted by the neural crest from the mesoderm [14]. Contrary to this, we rather support, also in line with previous ideas [21,26,55], a unique or convergent elaboration of additional ectomesenchymal neural crest fates on the basis of pre-existing neuroepithelial fates. Convergent elaboration means that additional fates can be independently developed using a similar or different molecular toolkit as compared to analogous phenotypic outcomes (cell types) from other origins. In one of their reviews, Clare Baker and Marianne Bronner-Fraser discuss at length the possibility of independent elaboration of numerous non-ectomesenchymal and ectomesenchymal fates in a proto-neural crest from a single neuronal fate [21,26].

Moreover, to support the idea that mesenchymal fates could be elaborated *de novo* in the neural crest, we point out that dentin is a true unique neural crest innovation [14]. Besides, teeth-like odontode structures (conodont elements) represent the first hard mineralized tissue found in the bodies of extinct jawless chordate conodonts [56-58]. There are views inferring that odontoblasts represent an evolutionary modification of the neuroglial fate, being former electroreceptors or sensory cells monitoring temperature and chemical changes. These receptors were shielded by a collagenous and proteinaceous matrix that was later mineralized [51,59-61] (extensively discussed in [21,26]). This hypothesis is supported by the fact that odontoblasts express mechano- and thermosensitive transient receptor potential ion channels (TRPV1, TRPV2, TRPV3, TRPV4, TRPM3, KCa, TREK-1) and voltage-gated sodium channels, contact pain fibers and generally facilitate pain sensation in the tooth [62,63].

Since bone is very similar to dentin (while odontoblasts are similar to osteoblasts) and also appears in vertebrates together with the neural crest [14], it is plausible to hypothesize that evolutionary development of bone is a consequence of further odontoblast fate transformation in the neural crest lineage. In this case, osteocyte and osteoblast fates could be co-opted from the neural crest by the mesoderm or convergently elaborated in the mesoderm much later.

An interesting example of possible convergent elaboration is provided by Brian Hall and Andrew Gillis in their review where they remarkably point out that cellular cartilage tissue from cephalopod mollusks is morphologically indistinguishable from vertebrate cellular cartilage [14]. They suggest that the corresponding gene regulatory network is, in fact, very ancient and predates the origin of chordates. Despite our high appreciation of this idea, we doubt the possibility that the stereotypical cellular cartilage-forming gene regulatory network already existed in a common ancestor of vertebrates and invertebrates, was lost and then miraculously reappeared in vertebrates with the same key players. We would rather expect that there are different molecular mechanisms governing the cartilage fate in cephalopods and chordates. In line with our reasoning, the authors suggest (as one out of several alternatives) that cartilage could have independently evolved many times as a result of parallel evolution in different taxa including brachiopods, annelids, mollusks and arthropods [14,64]. Additionally, Daniel Meulemans and Marianne Bronner-Fraser analyzed the expression of 11 *Amphioxus* orthologs of genes involved in neural crest chondrogenesis and, based on this, suggested that the cellular cartilage gene regulatory network and corresponding fate were finally assembled only in the neural crest during evolution [55].

To conclude, there are views reinforced by a wide array of data that support the possibility of elaboration of mesenchymal fates in the neuroepithelial lineage. On the contrary, currently there is no support for the idea that neuroepithelial (non-ectomesenchymal) fates were eventually elaborated from ectomesenchymal fates, especially given that most researchers agree that the proto-neural crest evolutionarily originated from neuroepithelial cells. Thus, we will consider neuroepithelial (basal non-ectomesenchymal) fates to be the most ancient and primordial in the neural crest cell lineage.

Notably, neural crest cells appear during development when the nervous system is generally assembled in the embryo, and multiple specialized (intrinsically or extrinsically) lineages of neuroepithelial cells exist. Which of these lineages could give rise to the neural crest in evolution, and how did this initial specialization impact the resulting repertoire of fates? How was this specialization important for the evolutionary scenario and driving forces of consecutive transformations? These are the questions we attempt to answer in this article.

Despite multiple recent advancements in the field of neural crest origin and evolution, lots of questions are still open because of the impossibility to trace the evolutionary history of the neural crest to the exact cellular origin in primitive chordates. Also, there is no evolutionary scenario that would explain why, for instance, neuroepithelial cells of pigmented lineage would transform into migratory multipotent neural crest. Thus, the main aim of the current article is to provide a testable hypothesis that would integrate current knowledge, solve molecular homology issues and obtain previously unexplored insights into the evolutionary history of the neural crest cells. Here we attempted to deal with this challenge and propose that first neural crest cells originated from the multipotent progenitor of the pigmented photoreceptors in an extinct lancelet-like organism. Below we will discuss molecular homology and other arguments supporting this point of view and will outline a potential evolutionary scenario of how the primitive pigmented photosensory structures could turn into the neural crest.

## Presentation of the hypothesis

### To see or to camouflage?

When we think about photoreception-specific traits, two quite unique functional features come to mind: photoreception and protection from indirect light by pigmentation. As we will see below, both of these traits are present in the neural crest lineage. For example, expression of a melanocyte-specific opsin (melanopsin), a photosensory protein, has been reported in teleost fish skin, chick melanocytes and dermal melanophores of *Xenopus laevis* in which melanopsin regulates the distribution of melanosomes in response to light [65-69]. The whole phototransduction cascade was found in melanopsin-expressing melanocytes of *Xenopus*: it appeared that TRP channels controlled by the phosphoinositide second messenger system mediate Ca^2+^ influx upon light exposure. The second messenger system is, in turn, activated by melanopsin through phospholipase C (PLC) and Gq-coupled receptors [67]. Another recent study conducted by Wicks and coworkers demonstrated that opsin-mediated photoreception and phototransduction drive early melanin synthesis in human melanocytes. The authors showed that melanocytes modulate the amount of melanin via a complex mechanism that includes activation of endogenous rhodopsin receptors by UV light followed by calcium mobilization through a G protein- and PLC-mediated pathway [70]. In line with this, Xue and colleagues demonstrated that melanopsin and PLC signaling operates in the iris, where they form the basis for the autonomous pupillary light reflex [71]. What is more, the photosensory cells in the iris, melanocytes and smooth muscle cells of iris sphincters are neural crest-derived, as has been shown in a chick-quail neural crest transplantation experiment [72]. The results of these studies clearly show the presence of visual photopigments and phototransduction cascades in melanocytes by several approaches ranging from functional experimentation to expression analysis studies. Thus, melanocytes can be regarded as tiny unicellular ocelli in the skin of vertebrates performing a number of non-visual functions. Apparently, these ocelli are born from the glial progenitors lining peripheral nerves during development and appear to be innervated in the adult skin [73-75].

Melanocyte-specific photopigments, melanopsins, are evolutionarily distant from the typical vertebrate opsins and belong to the group of rhabdomeric opsins – photopigments usually utilized in rhabdomeric photoreceptors of invertebrates [76-78]. There are two groups of photoreceptors found in nature: rhabdomeric (predominant in invertebrates) and ciliary (dominate in the eyes of vertebrates). Ciliary and rhabdomeric photoreceptors exhibit significant differences in the morphology of the membrane where the photopigments are packed and in the downstream signal transduction machinery. In ciliary photoreceptors, photopigments are incorporated into the membrane of a modified cilium, while in the case of rhabdomeric photoreceptors photopigments are inserted into the membrane folded into microvilli. When it comes to signaling, rhabdomeric photosensory cells utilize the PLC-based signal transduction cascade, get depolarized in response to light and are able to convert used rhodopsin (metarhodopsin) back into the active state by receiving another quanta of light. This is not true for ciliary photoreceptors, which rely on an external supply of 11-cis retinaldehyde in order to get back to the active state and use a phosphodiesterase-based signal transduction cascade, also hyperpolarizing in response to light [79,80]. Importantly, both types of photoreceptors share a common evolutionary origin in the distant past and in some cases might develop from the same progenitor cell, for example, in the vertebrate eye [81,82]. Consequently, expression of melanopsins together with a PLC-based phototransduction pathway suggests that neural crest-derived melanocytes are related to the invertebrate rhabdomeric photoreceptors.

Melanopsins are not exclusively found in neural crest-derived melanocytes. For example, melanopsins are also expressed in retinal ganglion cells of the vertebrate retina where they play a prominent role in setting up the circadian rhythms [83]. This discovery suggested that retinal ganglion cells are likely evolutionarily derived from ancient rhabdomeric photoreceptors [84]. In the most basal chordates melanopsins are found in rhabdomeric photoreceptors represented by the Hesse organs of *Amphioxus*, which consist of photosensory cells and photosensory melanin-containing cup cells [78,85]. The presence of a melanin together with melanopsin-mediated light sensitivity in the pigment cup cells of the lancelet strongly parallels melanocytes, which exhibit the same features. Could this highlight an unexpected evolutionary connection? Furthermore, the combination of pigmentation providing protection from indirect light with the ability to sense light in the same cell reflects an ancient and archetypal feature appearing in very basal photoreceptors [86]. Contemporary examples of such photosensory cells include not only vertebrate melanocytes and organs of Hesse, but also sponge larva photosensory cells, cnidarian planula photoreceptors and ascidian ocellus [85,87-89]. Thus, the lineage of pigmented rhabdomeric photoreceptors from the ancient lancelet-like animal, executing both photoreceptive and pigmentation programs, might be a possible origin of the proto-neural crest, since it resembles neural crest-derived melanocytes [77,85,90].

However, it appears that migratory neural crest-derived melanocytes also express rhodopsins of ciliary photoreceptors [68,70,91]. This fact represents a problem that is not easily reconciled with the previous logic. This suggests that melanocytes represent a mixed type of pigmented photoreceptor, and, thus, they cannot be derived from either of the known photoreceptor types on its own. On the other hand, the multipotent progenitors of the vertebrate retina give rise to both rhabdomeric (retinal ganglion cells) and ciliary photoreceptor cell types (rods and cones) together with neurons, glial and retinal pigmented epithelium (RPE) cells, comprising at least eight different cell types in total [82,92]. If such a multipotent progenitor cell of the complex ocellus could transform into migratory proto-neural crest, then it would be possible for some features of the lineages to merge, resulting in a novel functional chimerical cell, such as the melanocyte. To make this assumption complete, we imply that relatively simple ancestral protochordate photosensory structures already contained several distinct cell types, including ciliary and rhabdomeric photoreceptors originating from the same progenitor cell type located in the neural ectoderm. Indeed, Ted Erclik and coworkers argue that the eye of the common bilateralian ancestor already contained both ciliary and rhabdomeric receptors [93]. Additionally, Detlev Arendt suggests that during vertebrate evolution ciliary photoreceptors switched from a non-visual function to a visual function, while rhabdomeric photoreceptors did exactly the opposite in the same eye [94]. Contrary to this, all photosensory systems in *Amphioxus* are represented by either ciliary or rhabdomeric photoreceptors and not by their mixtures [95]. It renders the lancelet an imperfect model for identifying the precise photosensory structure that could give rise to the neural crest. Moreover, lancelets represent a side branch of the evolutionary tree of chordates and cannot be considered our direct ancestors. Still, we assume *bona fide* that the ancestral protochordate animals could possess relatively simple eyes or ocelli built and distributed like the Hesse organs of *Amphioxus* with one main difference: such structures must have already contained a mixture of ciliary and rhabdomeric receptors. Here we suggest that multipotent progenitors of such photosensory organs might have transformed and given birth to primarily multipotent proto-neural crest cells.

To further support the paralogous connection between the photosensory structures and the neural crest, we put forward the fact that the ocellus and the otolith are the only melanin-containing cells in the ascidian tadpole originating from the same a8.25 blastomere expressing the neural crest markers Pax3/7 and Snail [96]. Apparently, the sibling of the ocellus, the pigmented otolith, is able to delaminate almost completely from the cerebral vesicle wall into the lumen [97,98]. As Clare Baker put it in her insightful review addressing this: “if a melanin-containing cell arising from a Snail^+^ neuroepithelial precursor delaminated outside the cerebral vesicle, rather than almost delaminating into its lumen, we would call it a neural crest cell” [21].

In line with this, Philip Barron Abitua and Michael Levine with coworkers proved that the pigmented ocellus lineage in tunicates can be successfully reprogrammed into a migratory multipotent neural crest-like population by co-option of Twist, otherwise expressed only in mesodermal cells of *Ciona intestinalis*. Moreover, they demonstrated that the fate of a tunicate pigmented ocellus is controlled by Wnt signaling in a way similar to vertebrate neural crest and melanocyte specification [29,99]. MITF, a master regulator of a vertebrate melanocyte fate, also turned out to be expressed in a9.49 lineage together but not simultaneously with another transcription factor, FoxD. Importantly, tunicate FoxD was able to repress MITF in the lineage of pigmented ocellus similar to the molecular mechanism specifying neural crest-derived pigment cells in vertebrates [29,100,101]. Such conservation of specification machinery including FoxD and MITF strengthens the unexpected evolutionary connection between the pigmented photosensory lineage in protochordates and vertebrate neural crest.

Historically, MITF has been found to be associated with pigmentation, not only in the vertebrate eye and neural crest-derived melanocytes, but also in the ciliary eyes of cubozoan jellyfish *Tripedalia cystophora* – a very ancient and primitive diploblastic animal [102]. This gives the impression that MITF and its downstream program represent the module responsible merely for melanin-based shading and coloration conserved from ancient times. We believe that such an impression is at least incomplete. In fact, MITF is essential in the vertebrate eye, participating in the partitioning of the optic vesicle into the future RPE and retina. Later on, MITF defines the RPE cell fate and also drives pigmentation together with Otx2 [103,104]. Likewise, MITF is the master regulator of the neural crest-derived melanocyte fate and identity in the first place [75,105]. Interestingly, in the compound rhabdomeric eye of *Drosophila* MITF has a function not connected with the production of melanin or other pigments. Instead, it is expressed in the peripodial membrane and regulates the size of the developing neuronal photosensory part of the eye inside of the eye-antennal disc. This melanin-independent role of MITF during *Drosophila* eye development suggests its main function, the one conserved between the fly and the mouse, is that of fate restriction [106]. Furthermore, cutaneous melanin-based pigmentation in *Drosophila* is not regulated by MITF [107], and many protostome animals, despite having MITF, do not use melanin as a shading pigment in their rhabdomeric eyes, utilizing instead ommochromes and pterins [108]. In fact, there are no data confirming the role of MITF in cutaneous pigmentation (even melanin-based) outside of the vertebrate lineage. Thus, MITF is not necessarily connected to the production of the melanin, but is always operating during development of photosensory structures in studied proto- and deuterostome model organisms. The cases where MITF regulates pigmentation are most likely to be found in the eyes, for instance, in pigmented ocelli lineage of tunicates [29]. Interestingly, Clare Baker and Marianne Bronner-Fraser connected pigmented fate with protection of photoreceptive cells. The authors mention that once melanin-containing cells have evolved, they became useful for camouflage and are recruited to locations outside of the central nervous system [26,109]. Based on the mentioned arguments, we strongly suggest that the presence of MITF controlling melanin-based pigmentation is, in fact, a photosensory structure-specific trait. Therefore, we conclude that the presence of MITF in the neural crest lineage is indicative of an evolutionary connection between the latter and the ancestral photoreceptive organs.

Still, one of our general self-criticisms is that molecular mechanisms and cellular functions found in both the developing photosensory structures and the neural crest can be the result of a mere co-option. It is very tempting to suggest, for example, that recruitment of MITF expression into the neural crest lineage would enable the neural crest to produce migratory, pigmented melanocytes. The mechanism for such a change can be via a mutation in the regulatory regions of MITF providing a new ectopic site of expression. This event does not look improbable, and, obviously, such things must take place fairly often during evolution. However, recruitment of only MITF to a new ectopic location would most likely not result in the factual production of pigment, since MITF operates in the context of a network of transcriptional factors tailored to drive a pigment cell phenotype. The presence of Pax and SoxE transcriptional factors in addition to MITF is necessary to make functional melanocytes able to synthesize melanin [110-112]. This point of view is supported by the fact that MITF is also expressed in the developing vertebrate heart, mast cells and osteoclasts. These cell types are not pigmented, confirming that MITF alone is not sufficient to induce melanin synthesis [113-115].

Another argument includes that the pigmented fate in chordates is intimately connected to the photosensory fate, both in terms of transcriptional regulation and phylogenetic occurrence, as we discussed above. Based on this, we suggest that, in fact, pigmented and photosensory features can be united into one fate in the line of chordate animals. If we would anticipate a co-option of this fate from another tissue into neuroepithelial cells, we would need to point out such location or possible tissue of origin. However, in chordates, photoreceptors (pigmented and non-pigmented) are derived from the neural epithelium. Thus, we do not see the need for co-opting this fate into the proto-neural crest since it is already present in the neuroepithelial lineage.

To finally conclude this section, we suggest that the protochordate photosensory structures and the neural crest are deeply paralogous (and probably also in some cases orthologous) formations sharing a single evolutionary origin represented by the multipotent progenitor of the pigmented protochordate ocellus. This scenario implies that both pigmentation and photosensory programs evolved by switching their physiological role from light-based perception and circadian rhythm modulation to dynamic camouflage and protection from UV light in the newly born proto-neural crest lineage.

### Photoreceptor progenitors transform into the proto-neural crest cells: a hypothetical evolutionary scenario

For some time researchers considered *Amphioxus* to be an example of a degenerated evolutionary design [116]. However, this point of view did not find support later. Currently *Amphioxus* is thought to be a promising Evo-Devo model reflecting the early primitive condition of chordate organization [42,117]. Like many researchers before us, we attempt to find traces of the neural crest evolutionary origin in this basal animal model. Despite recent advances in identification of the rudimentary neural crest in ascidians, we do not see the evolutionary scenario of ocellus-to-neural crest transformation in tunicates because of their specific lifestyle and position on phylogenetic tree. On the other hand, we understand that the lancelets also represent a side branch of the chordate phylogenetic tree and are not direct ancestors of vertebrates. Still, we believe that extinct ancestral protochordates resembled *Amphioxus* in many ways, although their eyes might have contained both ciliary and rhabdomeric photoreceptors. Since we propose that neural crest cells originated from progenitors of photoreceptors in a lancelet-like central nervous system, we will briefly discuss how sensory systems are organized in the *Amphioxus*.

The lancelet has four morphologically and most likely functionally distinct photoreceptive modules. All of them are immersed in the CNS and include a frontal ventromedial eye built of ciliary photoreceptors, a lamellar body positioned dorsally in the anterior part of the neural tube (also composed of ciliary photoreceptors), rhabdomeric photoreceptors called Joseph cells forming a dorsal column and finally numerous rhabdomeric Hesse organs (dorsal pigmented ocelli) positioned in hundreds in the ventral neural tube along the whole body length [95]. The frontal eye and lamellar body are thought to be homologous to the paired vertebrate eyes and pineal organ, respectively. The frontal eye regulates the orientation of the lancelet larva when it feeds at the water surface [95,118]. Thurston Lacalli suggested that the lamellar body controls the circadian rhythms and vertical migrations of *Amphioxus* larvae living in deep waters. Lacalli also proposed that Joseph cells with dorsal ocelli are likely involved in monitoring the vertical position of the *Amphioxus* in a burrow since some ocelli may gradually become shaded by the substrate of the sea floor. Hesse organs and Joseph cells are positioned along the anterioposterior axis of the body and in this way resemble neural crest cells delaminating from the neural tube all the way from head to tail [95].

In addition to photoreception, lancelets possess numerous primary sensory neurons in various peripheral tissues (including the epidermis and different body cavities) and secondary sensory neurons located inside the central nervous system [42,95]. Intramedullary secondary sensory neurons appear to be somewhat similar to Rohon-Beard neurons and the sensory neurons of dorsal root ganglia in vertebrates. Two main types of these neurons are identified: Retzius bipolar cells and dorsal root cells, both positioned in the dorsal aspect of the neural tube. The function of these neurons is suggested to be mechanoreception in various peripheral locations where these neurons project [119]. For more details on the primary sensory cells in chemosensory and olfactory reception, oral innervation, epithelial tactile reception and other features of the peripheral nervous system, please see [95].

**Figure 3:**
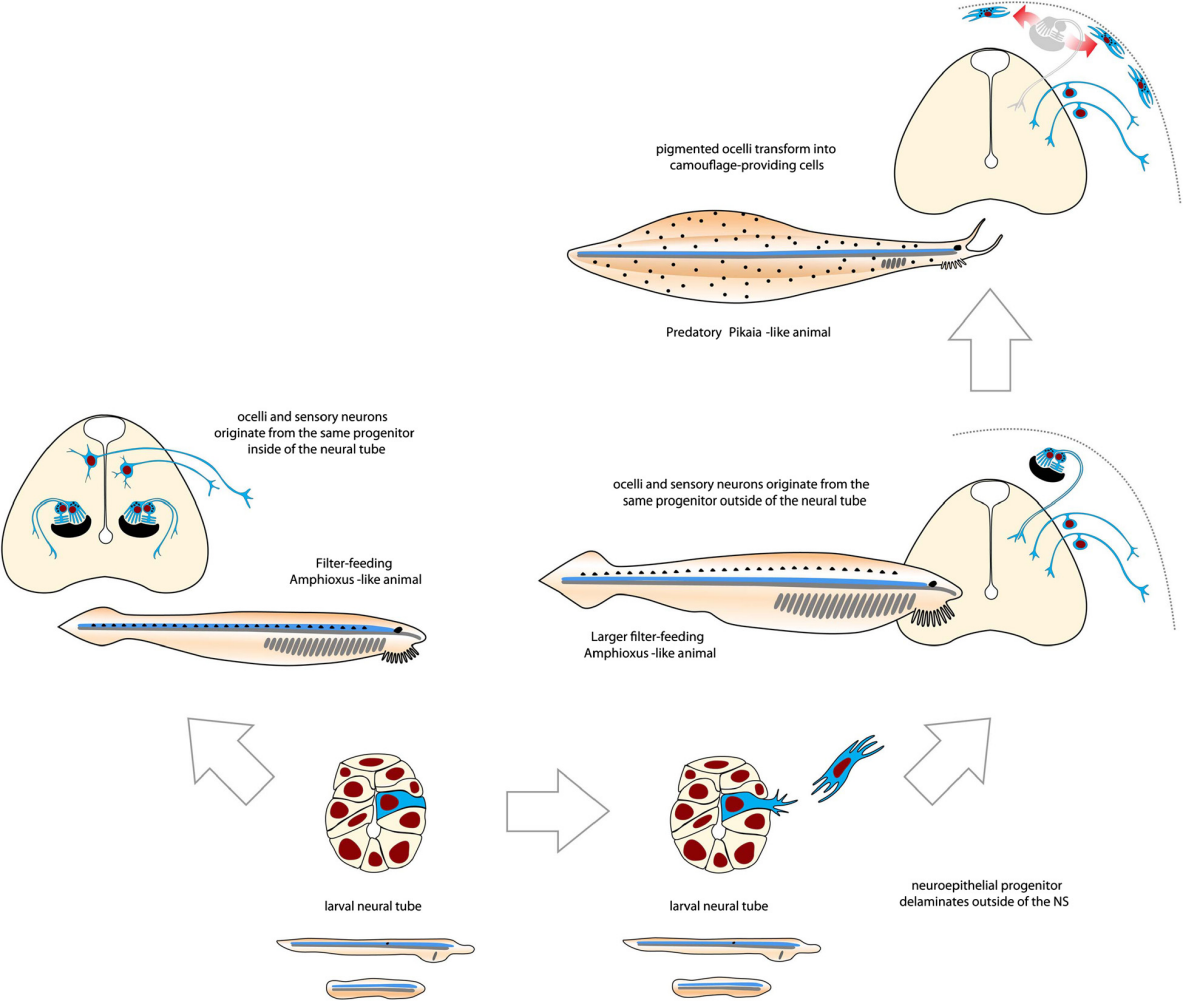
Evolutionary scenario outlining how common precursors of small pigmented ocelli and sensory neurons gave rise to neural crest cells in a lineage of early chordate animals.

Apparently, many evolutionary innovations are supposed to occur during embryonic development on the level of multipotent progenitor cells. Interestingly, pigmented and photosensory cells, secondary sensory neurons and glia from the adult lancelet-like CNS can be traced to one developmental origin – some multipotent progenitor in the early neural ectoderm. If these progenitor cell could gain the ability to migrate outside of the neural tube and produce the same repertoire of progeny at the periphery, this would essentially produce a proto-neural crest cell. Here we suggest how Hesse-like ocelli progenitors might have transformed step by step into migratory multipotent proto-neural crest stem cells during the early evolution of chordates (Figure 3).

Firstly, our evolutionary scenario suggests the repositioning of the Hesse-like ocelli from the inside of the central nervous system to subcutaneous locations. Although the dim light at the ocean floor efficiently penetrates the thin body of the lancelet down to the photosensory cells inside of the neural tube, some ancient chordates might have acquired larger body size to avoid the press of predation, requiring the photosensory cells to reposition themselves closer to the body surface in order to detect enough light, preferably under the ectoderm. For this purpose the ocelli progenitor cells must have developed an ability to migrate out of the neural tube and differentiate into the pigment and photosensory cells at the periphery. Such repositioning of the multipotent neuroepithelial eye progenitors could possibly have an interesting side effect: if this progenitor was so basal that is was also giving rise to the secondary sensory neurons (similar to Retzius and dorsal root cells), the cell bodies of those might have been transferred to the peripheral location together with accompanying glia from the same lineage. This scenario is similar to the hypothesis proposed recently by Fritzsch and Northcutt, and also by Philip Donoghue, Anthony Graham and Robert Kelsh, in which precursors of Rohon-Beard sensory neurons migrated out of the neural tube and gave rise to the peripheral progeny consisting of sensory neurons and glia [13,50]. Apparently, our hypothesis reconciles both points of view and goes further to reconcile an additional hypothesis partially discussed by Clare Baker, suggesting that protochordate peripheral glial cells might be related to vertebrate Schwann cells [21] and thus might represent primitive neural crest-like cells in our opinion. Indeed, rare axon-ensheathing glial cells have been reported to localize at the dorsal roots of the adult *Amphioxus* animals [120,121]. The function of these cells is unknown, and it is unclear whether these cells are able to migrate and give rise to any other cells type. In our opinion these rare glial cells strongly resemble invertebrate peripheral glia found in *Drosophila* and other protostomes [122,123]. On the other hand, recent studies on the vertebrate neural crest-derived tissues showed that early peripheral glial cells (Schwann cell precursors) give rise to melanocytes while staying inside the nerve. Literally, pigment cells are born on the nerve surfaces in localized clusters. This process, operating in mammals, birds and teleost fishes, is evolutionarily conserved [74,75,124-126]. Furthermore, cyclostomes appeared to have pigmented cranial nerves [127]. This connection between the nerve, glia and pigmentation highlights that ancient peripheral glia-like cells could be the multipotent progenitors emigrating out of the neural tube and giving rise to the pigmented subcutaneous ocelli, glia of the connecting nerves and probably peripheral sensory neurons.

Next stage of our evolutionary scenario proposes a loss of the importance of these lateral subcutaneous pigmented eyes to environmental awareness and in control of body position in the burrow. This could be the result of the already acquired relatively large body size stimulating progressive cephalization and sensory advancements in the anterior segment. Indeed, recent paleontological findings uncovered extinct primitive cephalochordates, for instance, *Pikaia gracilens,* which were larger than lancelets, were equipped with a developed “head” bearing appendages, a high flat body and fins – all features pointing to active locomotion and possibly predation [128,129]. Anterior eyes of such animals could proficiently master visual function, providing cues for successful navigation, swimming, attacking prey, avoiding predators and thus, finally, moving away from the filter-feeding lifestyle. The aforementioned changes could lead to the eventual functional degeneration of the lateral subcutaneous ocelli intended to provide the feedback on the position in the burrow. However, the photosensitivity and the ability to produce melanin-based pigment were likely retained in the lateral ocelli lineage allowing light-controlled pigmentation of the body and protection against UV radiation.

Thus, we propose that ancestral protochordate animals might have possessed numerous multicellular ocelli distributed along the anterior-posterior axis like those we see today in *Amphioxus*. These ocelli originated from the basal multipotent neuroepithelial cells in the neural tube. This early neuroepithelial progenitor could also give rise to the glial and secondary sensory neuronal lineages. Changes in the lifestyle and photoreception needs firstly repositioned these ocelli from the neural tube to subcutaneous locations by triggering migratory behavior of the basal neuroepithelial multipotent progenitor cell. It might have led to a novel placement of some secondary sensory neurons and glia outside of the embryonic neural tube. Later, in a course of cephalization, the lateral ocelli degenerated, switching their main function from seeing to conceiving and thus gave rise to the pigmented and photosensory components of the proto-neural crest. Further modifications of the migratory multipotent neuroepithelial precursors included slow and eventual elaboration of additional fates by some unknown molecular mechanics providing co-options of existing mesodermal and other neuroglial properties [14,21,55,130-133].

An alternative scenario (suggested by Clair Baker, personal communication) implies that progenitors of Joseph photosensory cells represent the likeliest evolutionary origin of the vertebrate neural crest since Joseph cells are positioned more dorsally as compared to Hesse organs and thus might share the lineage with dorsal bipolar cells (intramedullary sensory neurons). This situation can be compared to the actual lineage relationship between the Rohon-Beard sensory neurons and neural crest in fish and amphibian embryos, which adds additional weight to such scenario [13,26,134-136]. However, in the case of such alternative scenario it is unclear how the pigmentation program was recruited into the proto-neural crest. Additionally, given the latest discovery regarding the relation of the ascidian pigmented ocellus lineage to the vertebrate neural crest [29], it seems that pigmentation and expression of MITF were the basal and the most ancient properties of proto-neural crest lineage. Notably, the expression of MITF and presence of melanin have not been discovered in a Joseph cell lineage so far. On the other hand, the embryonic neural tube of *Amphioxus* is an incredibly small structure compared to the neural tubes of other vertebrate embryos [95]. Therefore, the exact dorsoventral position of progenitor cells giving rise to the proto-neural crest might not be so important, especially taking into account the absence of any information regarding the dorsoventral position of Hesse organ progenitors in the *Amphioxus* larva.

## Testing the hypothesis

In order to choose between the alternatives, it is essential to define the lineage relationships between different neuroepithelial progenitors and resulting photoreceptors, pigmented cells, secondary sensory neurons and peripheral glia in the developing *Amphioxus*. To achieve this we suggest elaborating a lineage-tracing system based on microinjection or selective genetic recombination. Successful identification of a common progenitor generating all above-mentioned cell types will provide strong support for our hypothesis.

Finally, we encourage increasing our knowledge of the possibility and mechanisms of co-option of whole gene regulatory networks, their parts and individual genes. Such knowledge will be a cornerstone of our understanding of the origin of the neural crest and its evolution.

## Implications of the hypothesis

To sum up, our scenario suggests the orthology of multipotent neuroepithelial progenitors of the ocelli from ancestral protochordate animals to the vertebrate neural crest cells. Apparently, changes in photoreception needs might have been the driving force behind the emergence of the proto-neural crest during the course of evolution. We propose that proto-neural crest cells were born as already multipotent population giving rise to the neural, glial, photosensory and pigmented progeny. During further evolution, the repertoire of fates was enriched by slower co-options of mostly mesodermal features and functions. We believe that our reasoning does not deny but rather extends a number of existing views on the neural crest origin and evolution. Like many other researchers, we root the origin of the proto-neural crest in some ancient neuroepithelial progenitor cell, with the additional refinement of narrowing down what kind of neuroepithelial progenitor this cell could be. This knowledge is specifically important for our understanding of the origin of novel cell types during evolution of multicellular organisms – one of the key questions of modern biology. If our hypothesis is true, we expect to gain an essential insight into how new fates and properties are elaborated in the neural crest lineage, which would allow further experimental manipulations and reprogramming of cell lineages for the sake of fundamental science and human health.

## Abbreviations

CNS: Central nervous system; PNS: Peripheral nervous system; PLC: Phospholipase C.

## Competing interests

The authors declare that they have no competing interests.

## Authors’ contributions

IA generated the original idea, drafted the manuscript and wrote the final text. EI participated in shaping the original idea, searching for supporting arguments and designing the logic of the manuscript. EI also drafted the manuscript together with IA. Both authors read and approved the final manuscript.
